# The League of Mercy

**Published:** 1908-12-26

**Authors:** 


### HOSPITAL ADMINISTRATION.

### CONSTRUCTION AND ECONOMICS.

### THE LEAGUE OF MERCY.

NINTH ANNUAL MEETING OF PRESIDENTS.

Letter from the Prince of Wales.

The ninth annual meeting of the Presidents of the League of Mercy was held in the Board Room of St. George's Hospital on Monday, December 21. Lord Farquhar (President of the West Marylebone District) presided.

Colonel H. B. Hamilton (Hon. Secretary to the Presidents) read the minutes of the last meeting and a telegram from Princess Victoria regretting her inability to be present.

Mr. E. W. Wallington (Hon. Registrar of the Order of Mercy) read the following letter from the Prince of Wales, the Grand President of the League:—

"I am directed by the Prince of Wales to say that the Princess and he are glad to learn from the reports which they have from time to time received that the year now closing has proved no exception to previous years in regard to the steady progress and expansion of the work of the League of Mercy.

"It is gratifying to their Royal Highnesses to know that the concert at the Albert Hall last May not only afforded a pleasant and enjoyable opportunity for gathering together large numbers of the officers and workers of the League, but also realised a profit of more than £700.

"As the Prince of Wales anticipated would be the case, and as the extension of the League into the Home Counties fully warrants, the grants to the hospitals lying outside the area of distribution made by the King's Fund in London have this year been increased 50 per cent.

"As Grand President and Lady Grand President, their Royal Highnesses desire once again to thank very heartily all workers of every grade who have contributed to these satisfactory results, and they are pleased to hear that with the accession of the new Organising Secretary, Colonel Kempster, there has been no falling off in the good work as the League enters on the tenth year of its existence."

Lord Farquhar said the letter which had been read from the Prince of Wales showed the deep interest their Royal Highnesses had always taken in the work of the League. The letter had alluded to the progress which had been made in the work and the increase of the subscriptions, and he desired to make it quite clear that if they wished to succeed in getting subscriptions in the Home Counties they must be liberal in their contributions to the hospitals. He desired also to mention the regret he felt at the resignation of Mr. Harold E. Beaumont, who for five and a half years had acted as Assistant Secretary, and for a short period as Secretary to the League, and had proved himself a very steady and hard-working official. Mr. Beaumont had been replaced by Col. F. J. Kempster, who promised to be a very able Secretary, and he was very glad to think that the League in the future would be guided by a man whom they felt to be very competent and efficient.

Another point to which reference had to be made was the concert which took place in May last at the Albert Hall. So far as his experience went it was one of the most successful concerts ever held, and he did not hesitate to say that so far as the League was concerned it was one of the most interesting events in its history. It was a most beautiful concert; the spectacle was extremely fine, and the result of it must have been most satisfactory, since by its means they had not only been able to give great pleasure to everyone who was present, but to hand over a sum of £700 to the credit of the fund. In thinking of those to whom the credit of the success of the concert was due they had to thank his Majesty the King and her Majesty the Queen, who proved their interest in the objects of the League by graciously attending. Their thanks were also due to their Royal Highnesses the Prince and Princess of Wales, who had supported it in every possible way. The Princess of Wales had presided at one or two meetings at Marlborough House in connection with the work before the concert. Princess Victoria had also presided at several meetings, and had encouraged in every possible way those who were attending to details of the work. Princess Louise had also assisted them, and altogether the League were under a deep debt of gratitude to the Royal Family. The next people to be remembered were those who took part in the concert. The entertainment was organised by Signor Paolo Tosti, while Madame Melba and Signor Caruso sang. He could imagine no finer purpose to which a beautiful voice could be put than that of charity, and they were deeply grateful to all the artists. Their thanks were also due to Mrs. Harrison, Lady Dimsdale, and Mr. Wallington. (Cheers.)

He was glad to say that the position of the League and the last year's working were very satisfactory. He was certain that if all the members of the League would take an example from the organisation and hard work which had been displayed during the year, and from the concert which took place in May, he had no doubt that next year they would have a collection of £25,000, and he hoped that would be the case.

Mr. F. S. W. Cornwallis moved "That Lord Farquhar be appointed chairman, Mr. Frederick Green deputy chairman, and Colonel H. B. Hamilton hon. secretary."

The resolution was in the same form as it was last year, and as he hoped it would be for many years to come.

Sir Walter Johnson, in seconding, said Lord Farquhar had been too modest as to his share in the work of the year, but the workers in the movement knew the advantage they derived from the fact that Lord Farquhar was their chairman, and knew how to appreciate his services.

The resolution was adopted.

Sir Fitzroy D. Maclean moved that the following seven members be appointed to the Executive Council:—Lord Farquhar, Mr. R. C. Antrobus, Mr. Frederick Green, Mrs. R. L. Harrison, Mr. Edward Murray Ind, Lady Morrish, and Mr. Montague Sharpe.

The resolution was also agreed to.

A Record Year.

Sir Henry Burdett (the Hon. Treasurer), in submitting the annual financial statement of the year's work, said the Chairman had been far too modest. No one would ever have thought from hearing his speech that the League owed much to his wife and himself. The truth was that, if it had not been for Lord and Lady Farquhar there would have been no concert, and he believed that the idea of the concert originated with Lady Farquhar. The concert had achieved far more extended and far deeper results than mere monetary value. It brought together those who were associated with the League of Mercy, and he had no doubt that this had had a material influence for good, the fruit of which would be shown in future years. If it had not been for the concert the League, having been at some disadvantage on account of changes in the staff, might have shown a falling off during 1908. He thought, however, that the meeting would agree with him in saying that the returns collectively were very satisfactory. Still, returns showed that in 52 divisions they were at the moment £5,166 short, while 39 divisions had raised £2,103 more, showing a net decrease of £3,063. The decrease in the figures was accounted for by the fact that sums amounting to £3,737 had not yet been received from certain districts which had promised them.

The Chairman: But they will come?

Sir Henry Burdett said he had every faith that the money would be sent in, but the treasurer, being a man of figures and not of faith, had to deal with what he had in hand. (Laughter.) The actual result was that the total collections, including promises, so far as he had been able to make them out, amounted to £22,100, the largest sum which had ever been received by the League. The returns from the various districts were as follows:

The Berks, Bucks, Hants, and Oxford Division had not paid in the sum raised as yet, but last year they contributed £2,108.
South Kensington, which contributed £1,313 last year, had sent £1,600, an increase of nearly £300.
Fulham, which gave £1,294 last year, gave £923, a decrease of £371. This was mainly caused by the transfer of a large sum from Messrs. Woodland's from this Division to South Kensington.
South Paddington had sent £774, as against £842, a decrease of £78.
Marylebone East £611, as against £543, an increase of £68.
Marylebone West £652, as against £367, an increase of £285.
Epsom and Esher £581, as against £622, a decrease of £41.
Kingston £504, as against £532, a decrease of £28.
Chelsea £462, as against £481, a decrease of £19.
Sevenoaks £452, as against £491, a decrease of £39.
North St. Pancras £423, as against £460, a decrease of £37.
North Kensington £386, as against £287, an increase of £99.
East Sussex £382, as against £40, an increase of £342.
Westminster £316, as against £316.
Hornsey and Finchley £313, as against £274, a decrease of £39.
Hampshire £313, as against £343, a decrease of £31.
Strand £297, as against £360, a decrease of £63.
Brentford £291, as against £182, an increase of £109.
Chertsey £271, as against £282, a decrease of £11.
Middlesex £267, as against £237, an increase of £30.
North-East Sussex £263, as against £150, an increase of £113.
Guildford £255, as against £265, a decrease of £10.
Hertford £236, as against £249, a decrease of £13.
Lewisham £233, as against £268, a decrease of £35.
Tunbridge £224, as against £272, a decrease of £48. £63 more had been promised.
Eye £222, as against £212, an increase of £10.
Dulwich and Norwood £212, as against £305, a decrease of £93.
Richmond £211, as against £182, an increase of £29.
Eastbourne £210, as against £168, an increase of £42.
Uxbridge £206, as against £214, a decrease of £8.
Ashford £196, as against £122, an increase of £74.
Luton £196, as against £177, an increase of £19.
Brighton £194, as against £200, a decrease of £6.
Epping £190, as against £195, a decrease of £5.
Woolwich £188, as against £178, an increase of £10.
Ealing £183, as against £212, a decrease of £29.
Deptford-Brockley £176, as against £305, a decrease of £129. But £70 more were promised.
West St. Pancras £175, as against £90, an increase of £85.
East Grinstead £166, as against £116, an increase of £50.
Brixton £162, as against £135, an increase of £27.
Horsham £158, as against £138, an increase of £20.
Maldon £156, as against £159, a decrease of £3.
Reigate £135, as against £103, an increase of £32.
North Hackney £139, as against £120, an increase of £19.
Mile End £129, as against £139, a decrease of £10.
Romford £125, as against £175, a decrease of £50. An additional £10 were promised.
Medway £124, as against £157, a decrease of £33.
City of London £122, as against £142, a decrease of £20. Lady Dinsdale was about to take charge of this Division, and he had no doubt the contribution would be substantially increased as, of course, it ought.
Greenwich £119, as against £133, a decrease of £14.
Battersea £118, as against £81, an increase of £37.
Bermondsey £112, as against £105, an increase of £7.
The increases shown in the two last mentioned poor neighbourhoods were very satisfactory.
Biggleswade £101, as against £119, a decrease of £18.
Croydon £90, as against £117, a decrease of £27.
Hythe £84, as against £67, an increase of £17.
Isle of Thanet £82, as against £101, a decrease of £19, with £15 promised.
Streatham £78, as against £77, an increase of £1.
West Islington £77, as against £102, a decrease of £25.
Lewes £76, as against £66, a decrease of £10.
Whitechapel £75, as against £75.
Lowestoft £73, as against £61, an increase of £12.
Limehouse £70, as against £44, an increase of £26.
Wimbledon £70, as against £70.
North Paddington £63, as against £112, a decrease of £49.
Lambeth £62, as against £5, an increase of £57.
Watford £62, as against £55, an increase of £7.
Hammersmith £56, as against £215, a decrease of £159, but with the promise of £159.
Worthing £54, as against £60, a decrease of £6.
St. Albans £50, as against £35, an increase of £15.
Harwich £47, as against £58, a decrease of £11.
Sudbury £47, as against £36, an increase of £11.
Rochester £44, as against £62, a decrease of £18.
North Camberwell £42, as against £44, a decrease of £2.
Faversham £39, as against £32, an increase of £7.
Bethnal Green £31, as against £27, an increase of £4.
St. George's £31, as against £964, a decrease of £933, with £910 promised.
Peckham £29, as against £37, a decrease of £8.
Walthamstow £29, as against £39, a decrease of £10.
Clapham £26, as against £28, a decrease of £2.
Albert Hall Concert, £738.
Scala Theatre Concert, £205.

In reading through those lists the members of the League will be conscious that during the present year the work in some of the divisions must have been discouraging. Nevertheless the record was a good one and the thanks of the League were heartily due to those workers by whose united efforts the fund had been raised to a higher figure than ever before. The expenses of the League were growing less instead of larger, and the outlook was a very encouraging one. The grant to the King's Fund would be £19,000, or £1,000 more than last year. The total distribution from the funds of the League during the year 1908 amongst the hospitals amounted to £20,516.

Sir William Collins, M.P. (one of the Hon. Secretaries), in describing the work of the League during the year said that the total distribution of money by the League during its nine years' existence had been no less than £119,595. The Prince and Princess of Wales had during the past twelve months appointed the following Presidents and Lady Presidents: Battersea, Lord Wandsworth; Camberwell and Peckham, Mr. Henry C. Gooch, M.P.; Lambeth and Kennington, the Rev. W. H. and Mrs. Hornby Steer; North Kensington, Mr. Alan H. Burgoyne; Brighton, Mrs. Pigeon; Dartford, Mr. and Mrs. Lewis P. Kekewich; Medway, Mrs. Best Dalison; Tonbridge, the Lady De L'Isle and Dudley. The Prince and Princess of Wales had also entertained 800 or 900 workers of the League at a garden-party at Marlborough House and awarded to forty-eight ladies and gentlemen the "Order of Mercy"—an order which is never given except for personal service. It was sometimes stated that the League was not sticking to the original intention of attracting the small subscribers. This was a mistake. One Vice-President of a poor district had written to say that £19 12s. 3d. of the sum sent had been subscribed by 4,000 poor people in penny subscriptions. He found that the poor people would give readily according to their means if they were made to understand that their contributions were really needed, and that they were directly without any deductions to the hospitals for which they were intended.

A Story and some Suggestions.

Mrs. Mateo Clark said she was sure they must all feel intensely gratified to hear that their League had again had a successful year. Those hours when friends had proved unsympathetic, and turned a deaf ear to suggestions that they should join the League, or when perhaps their favourite member had suddenly resigned, or when money had proved unobtainable from all and everyone—then they might, for a time, grow faint-hearted. But then, too, they knew that nothing in this world was obtainable without a certain amount of hard work, and so, even if they hesitated, most of them regained their courage very quickly and continued what was, after all, a labour of love. Sometimes, when she had felt a little disappointed, she had heartened herself again by the recollection of a little incident which occurred in a town in Spain she was visiting. Opposite the hotel was a large café, and into it there entered one day two Sisters of the Poor begging alms for their old people. They were such modest shrinking women that she was sure no duty could be more distasteful to them than this—yet they had to do it. They approached a group of noisy workmen who were gambling and drinking, and as the sister timidly placed her collecting-box upon the table, one of the men—infuriated probably by drink and losses—sprang up and struck her in the face. Angry and ashamed, some of the bystanders went to her assistance, but the sister waved them away, and, quietly wiping the cheek still reddened by the blow, she said gently: "That, sir, was for me; now what will you give me for my poor people?" After the good news they had heard that day they would feel both encouragement and satisfaction. If they considered the League with due care she thought they would come to the conclusion that it had before it the possibilities of becoming a powerful support to hospitals—not only London ones but in time also to many in the country. They had an advantage over other charities in that they did not remain stationary; their branches were growing and increasing all over the town, in the suburbs and down into the country. They were carrying their petition for help into every part, amidst every class. They passed from some town hall in the East End to a West-End mansion, from a suburban villa to a country house. No place was too important, no place too poor, for them to enter. In this way they were spreading the knowledge of how greatly the help of every single person was wanted—be they rich or poor, millionaire or workman, be their offering pounds or pence, they were trying to bring home to all that it was not alone a voluntary act but an absolute duty for them to support the hospitals. They would agree that they were doing good and useful work, and not alone in the way already mentioned but also in another way. They were acting as a link in that great chain of humanity which binds all classes together in questions of illness and suffering. They were trying to make the poor understand how readily their richer brothers give not only their money but their personal service to assist them. They were showing, too, how willing the poor were to give whatever small sum they could afford if only they realised it was really required. Personally, as one who studied human nature with some curiosity, she had found this work so intensely interesting that she had been more than amply repaid for any small trouble taken. She would like to offer one or two small suggestions which might perhaps be of use. She thought that, in the country, the town-hall meeting was a little lost sight of. Most of the meetings were held in large country houses and in the afternoon. They all knew that it is almost impossible for tradespeople and working people to leave their work in the afternoon, and also that they were often shy about venturing among county families. Evening meetings where a speaker could explain the objects of the League and ask their assistance, followed possibly by a small concert, would prove advantageous to the cause. She would also like to beg all Presidents or Vice-Presidents who were holding drawing-room meetings to try and invite, as well as those belonging to the League, as many strangers as they possibly could, for they were constantly in want of new recruits. People in the country did not always appreciate them nor realise their reasons for asking them for help, but this misunderstanding was gradually being cleared away, and country hospitals would find that, in time, they would become good and valued friends. But county districts must do two things. They must help towards success—and that most of them were already doing, for they had to thank and congratulate many of them most heartily for the capital work they had done this year. The other thing was that they should be patient and give the League time to gain in strength and power before they pressed too hardly for increased assistance. To whatever hospital they gave support, it was always good and useful work, for they were the foremost schools in the whole world. They taught human sympathy. What quality was there not shown in a hospital? Patience, devotion, unselfishness—there was not one single good trait in the nature of mankind which was lacking. Not only did they give back health and strength to the poor sufferers, but they gave them courage to face life again after they had experienced the love and care of whose very existence years of hardships and disappointment might have made them doubtful. Surely it must be pride and happiness to use the power which lay in their hands to assist in keeping up these homes of peace for those in pain. What matter the trouble? It was a duty which lay close to their hearts. Let them think what a satisfaction it was to know they had done a little towards helping those less fortunate than themselves. Their Christmas would be all the happier for the remembrance of what they had tried to do, and when the New Year came they would take up their work with fresh resolution to do their best, keeping the words ever before them: "Where we cannot find a way we must make one."

Theatre Benefit Performances: Their Use and Abuse.

Mr. George Alexander said he had been asked a few days previously if he would say something about what the members of his profession had done for charitable work in London during the past year, and on looking into the subject he felt a good deal of pride, because he had found that during the past two years ten London theatres had by special performances raised no less than £14,000. Something had been said about the value of personal service, and he thought that the members of the dramatic profession could claim that they at least were giving some personal service, and while they did all they could for outside charities they did not forget their own. Their poor were well looked after, and he thought it was a very great credit to the members of his profession that if they made money out of the public they did not forget to leave by their wills very large sums to their own charities. He and other members of the dramatic profession would be very glad to receive any suggestions from any of the Presidents as to how to increase the good work which was being done by the League of Mercy. He had lately undertaken the presidency of the South St. Pancras Division, and he hoped during the ensuing year to do some work in that quarter. They would be surprised if they knew how many applications were received by the managers of London theatres on behalf of charity, but his experience was that there was nothing that was so much abused as the charity performance—(hear, hear)—and he now made it a matter of business to discover what was the real motive for such applications. Sometimes it is a poor actor, sometimes a fond mother who had a budding Juliet in her brood; sometimes a dramatist who had sent a one-act play to the manager of every theatre in London; but he now made it a rule that those who were getting up the performance should guarantee, as the League guaranteed, the expenses of the entertainment so that the money paid at the doors should go directly and without deduction to the charity for which it was intended. He knew of one performance during last year at which the money received amounted to £562, but the charity only received £90. At another performance, on the other hand, the tickets realised £1,400 and the expenses were only £20. (Cheers.) He would warn Presidents of the League and those associated with them to be very careful in accepting performances until they knew that they were really legitimate and that the money would be paid to the charity. When some people took tickets for a performance on behalf of this or that hospital they seemed to feel that they had done all they could be expected to do for hospitals that year, whereas if they knew the whole of the circumstances they might find that the hospital had benefited to the amount of only a few pounds. If the League at any time made any suggestion to his profession he was quite sure members of that profession would regard it as a privilege to do anything they could to assist the League of Mercy.

### Votes of Thanks.

Dr. Strong moved and the Rev. W. H. Hornby seconded a vote of thanks to the authorities of St. George's Hospital for the use of the board room, which was carried with acclamation.

Dr. Scott moved and Mr. H. W. Keay seconded a vote of thanks to the Chairman.

Lord Farquhar, in replying, mentioned their indebtedness to Sir Ernest Cassel, who had sent his private orchestra to play before and after the meeting.

